# Maximum Aerobic Capacity of Underground Coal Miners in India

**DOI:** 10.1155/2011/232168

**Published:** 2011-09-25

**Authors:** Ratnadeep Saha, Netai Chandra Dey, Amalendu Samanta, Rajib Biswas

**Affiliations:** ^1^Department of Physiology, National Medical College, Bhediyahi-18, P.O. Box 78, Parsa, Birgunj, Nepal; ^2^Department of Mining Engineering, Bengal Engineering and Science University, Shibpur, Botanic Garden, West Bengal, Howrah 711103, India; ^3^Department of Occupational Health, Ergonomics and Human Performance Research Laboratory, All India Institute of Hygiene & Public Health, (A.I.I.H & P.H), 110 C.R. Avenue; Kolkata 700073, India; ^4^Department of Physiology, Himachal Dental College, Sundernagar, Himachal Pradesh, Mandi 175002, India

## Abstract

Miners fitness test was assessed in terms of determination of maximum aerobic capacity by an indirect method following a standard step test protocol before going down to mine by taking into consideration of heart rates (Telemetric recording) and oxygen consumption of the subjects (Oxylog-II) during exercise at different working rates. Maximal heart rate was derived as 220−age. Coal miners reported a maximum aerobic capacity within a range of 35–38.3 mL/kg/min. It also revealed that oldest miners (50–59 yrs) had a lowest maximal oxygen uptake (34.2 ± 3.38 mL/kg/min) compared to (42.4 ± 2.03 mL/kg/min) compared to (42.4 ± 2.03 mL/kg/min) the youngest group (20–29 yrs). It was found to be negatively correlated with age (*r* = −0.55 and −0.33 for younger and older groups respectively) and directly associated with the body weight of the subjects (*r* = 0.57 – 0.68, *P* ≤ 0.001). Carriers showed maximum cardio respiratory capacity compared to other miners. Indian miners VO_2max_ was found to be lower both compared to their abroad mining counterparts and various other non-mining occupational working groups in India.

## 1. Introduction

Assessment of maximum aerobic capacity or VO_2max_ has immense importance in the field of work physiology. It is the primary determinant of physical work capacity and reflects the functional efficiency of cardiovascular, respiratory, and neuromuscular systems of our body. Assessment of this parameter is of pivotal importance to determine the fitness criteria of a person engaged in any sort of activities.

Till date, studies reporting aerobic capacity of underground miners are available only from overseas literatures. Indian studies determining aerobic capacity of miners are restricted only for opencast metalliferous mines and studies of similar type among haulage-based underground coal miners still represent a virgin area to undertake.

In this scenario, an endeavour has been taken in the present study to assess the maximal aerobic capacity of underground coal miners. The data from present study may be helpful in evaluating the required level of physical fitness for working in various categories of mining activities for workers of different age groups and also to utilize their potential more effectively to increase mine productivity.

## 2. Methods

### 2.1. Selection of Subjects

Ninety-eight healthy miners from three different underground mines of West Bengal, India were selected following a random sampling technique stratified on the basis of the age. The subjects having a minimum work experience of five years were accustomed to work in heat. They had no report of medical history as confirmed by the respective health centers of the collieries. The miners selected were engaged in three different mining works, namely, drilling, shoveling, and carrying (Table 1). The choice of miners from these three categories of work was based on the fact that these works demand a significant working time at a stretch in the allocated working areas; secondly, these activities can be quantified in terms of work output and finally these activities were supposed to be physically demanding. They had a mean age of 41.3 ± 9.5 (23–58 yrs), and accordingly subdivided into two age groups, namely, <40 years (younger group) and ≥40 years (older group).

Before the study, interactions were carried out with the miners where they were explained about the objective of the study and the extent of their involvement. The selected miners agreed to render them voluntarily in accordance with the design of the experiment. Conventional Board and Pillar method of work were employed in these mines where depth of workings, degree of gassiness, seam thickness, gradient, seam water bearance, strata temperature, and humidity were seemed to be of similar type. 

### 2.2. Parameters Measured

Heights and weights of the subjects were recorded using an anthropometric rod and human weighing balance, respectively. Body mass index (BMI) [1] and body surface area (BSA) were calculated accordingly.

#### 2.2.1. Resting Heart Rate (HR_rest_)

It was measured by using lightweight telemetric equipment—Sport Tester PE 3000 (Polar Electro, Finland) at a regular interval of 1 min for a period of 20–30 minutes during their resting conditions. Maximal heart rate (HR_max_) of individual subjects was derived from age as 220−age [2]. Heart rate reserve (HRR) of the subjects was derived as the difference between the maximal and resting heart rate of the subjects.

#### 2.2.2. Maximum Aerobic Capacity: (VO_2max_)

It was determined indirectly through step test procedure as described by Martiz et al. [3]. Now in each time firstly they performed work with a stool of 30 cm high at a rate of 15 cycles/minute and then at a rate of 25 cycles/minute for a period of eight minutes; next with a stool of 40 cm high at a rate of 25 cycles/minute for a period of five minutes. The rhythm was maintained with the help of metronome. During exercise, heart rate and oxygen consumption in each workload were recorded at an interval of one minute. Oxylog-2 (P. K. Morgan England) was used to measure the oxygen consumption (VO_2_) of the subjects and the telemetric heart rate monitor to record the heart rate. 

For each subject, straight line equation was derived by method of least squares using heart rate as predictor and oxygen consumption as criterion variable. Finally VO_2_ was then extrapolated to the “age predicted maximal heart rate” to obtain the maximal aerobic capacity (VO_2max_) of the individual subjects which was expressed both in absolute terms (L/min) and in relative terms (mL/kg/min). All measurements were carried out in the surface in a comfortable rest area in the morning during 8.00–10.00 hrs before the beginning of the work shift. 

### 2.3. Analysis of Data

Descriptive statistics comprising mean as a standard index of central tendency, standard deviation as the measure of dispersion was used to present the physical and physiological criteria. The relationship between physical parameters (age and weight) and VO_2max_ was analyzed by using the product moment correlation coefficient (*r*). Linear models with physical characters as the predictor variable and VO_2max_ as criterion variable had been developed. *R^2^* value was considered to assess the degree of association between two parameters. Comparison between parameters was performed by means of test of significance and the *P* value was specified case wise. ANOVA (at *α* level of 0.05) was also done to find out any significant differences of different physiological parameters amongst various categories.

## 3. Results

Tables 2 and 3 summarized the physical and physiological characteristics of the miners in relation to different mining activities. It was evident from the tables that the miners did not differ significantly in terms of all the parameters studied except for weight where the driller group showed maximum value. The group also showed maximum absolute and relative VO_2max_ values in comparison to the other two categories.

### 3.1. Relationship between VO_2max_ with Age and Weight

**Figure 1:**
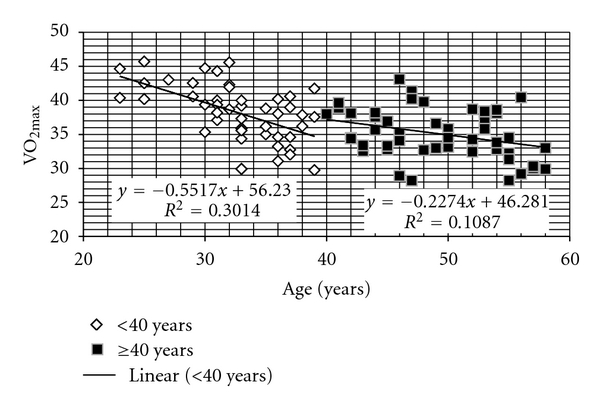
Relationship with VO_2max_ and age in younger (*n* = 47) and older (*n* = 51) groups.

The relationship between relative VO_2max_ (mL/kg/min) with age in two different age groups was depicted (Figure 1), and the results were summarized in Table 4. 

**Figure 2:**
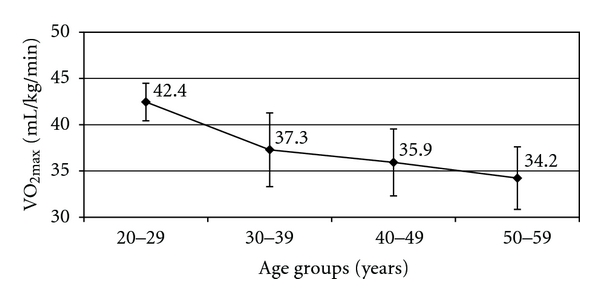
Mean VO_2max_ in relation to entire age group of the miners (*n* = 98).

A significant negative correlation was obtained with both the age groups. The average value of maximal oxygen uptake (mL/kg/min) in older group (35.1 ± 3.6) showed an 8.1% decline as compared to the younger age group (38.2 ± 4.2) whereas it showed a 6.45% reduction when expressed in L/min (Table 5). The average VO_2max_ (mL/kg/min) for the entire group of miners classified into different age groups was depicted in Figure 2. A conspicuous reduction of 12.2% in the mean value of the parameter was observed from “20–29” to “30–39” groups compared to much less decline of 3.7% from “30–39” to “40–49” group and 4.7% from “40–49” to “50–59” age group.

**Figure 3:**
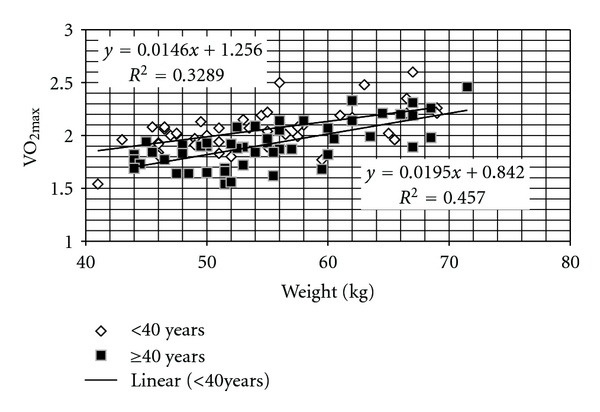
Relationship with VO_2max_ and body weight in younger (*n* = 47) and older (*n* = 51) groups.

Individual values of maximal oxygen uptake of two different age groups were also related to the body weight of the subjects and were depicted in Figure 3. 

Two different regression equations were computed for predicting the maximal oxygen uptakes (L/min) from weight of the miners in different age groups. The coefficients of regressions, standard errors of estimates, and *P* values of the regressions are presented in Table 5. It was evident that a high degree of positive correlation did exist at a high level of significance in case of both younger (<40 years) and older (≥40 yrs) miners.

### 3.2. Comparative VO_2max_ with Other Mining and Nonmining Occupational Groups

**Figure 4:**
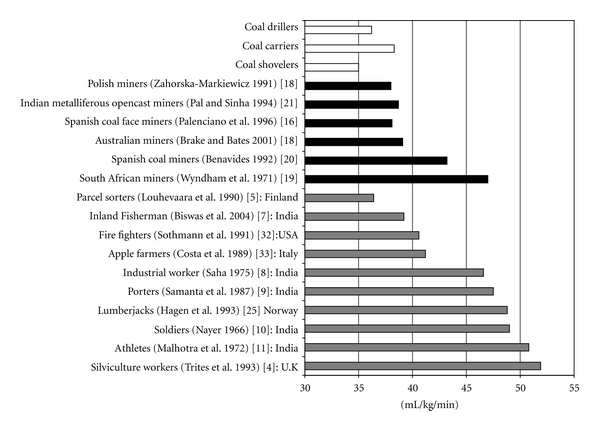
VO_2max_ of the underground coal miners (white rectangle) as compared to other miners (black rectangle) and nonmining occupational groups (grey rectangle).

Underground miners were inclined to have a lower than average aerobic capacity compared with general population and to other occupational groups. It was seen from the study that the average VO_2max_ of the miners was found to be 36.6 mL/kg/min which was not only below with respect to their fellow counterparts abroad but also found to be lower (5.7%) from their native working partners. It was the 2nd lowest VO_2max_ out of the other 16 values depicted in Figure 4 where the maximal value had been found in case of silviculture workers (51.9 mL/kg/min) [4]. The value in the present study was 41.8% lower than this group and only 0.5% higher than the average VO_2max_ of the parcel sorters (36.4 mL/kg/min) [5].

## 4. Discussion

The different category of underground miners who voluntarily took part under this study seemed to be maintaining homogeneity in terms of various physical and physiological characteristics except weight. However, the mean BMI for all categories showed that the subjects were typical of the average populations from eastern India [6].

### 4.1. Maximal Oxygen Uptake of the Miners

The average maximum aerobic capacity of the underground coal miners (36.6 mL/kg/min) obtained in this study had been lower than those for Indian inland fisherman, industrial workers, porters, soldiers, and athletes (39.2–50.8 mL/kg/min) in ascending order as depicted in Figure 4 [7–11]. So, the trend of lowered average aerobic capacity compared to general population and other occupations was in well concert with the results obtained by other investigators [12–15]. With respect to mining population abroad, the mean value was quite comparable with the Spanish [16], Polish [17] and Australian miners [18]. However, the aerobic capacities of South African miners (47 mL/kg/min) [19] and some other Spanish mine workers (43.2 mL/kg/min) [20] as found by other investigators was reported to be far higher than the observed value. The miners under present investigation showed a close similarity with the Indian metalliferous mineworkers [21].

 It was reported that VO_2max_ in general increases up to a certain age after which generally a steady gradual decline occurs [22]. In observation of Indian subjects, it was reported that maximal oxygen intake was influenced by age but significant decline was found to occur after 30 years of age [23]. Other studies in the same population reported a noticeable decrease of maximum aerobic capacity after 33 years of age both for industrial workers and porters as well [8, 9]. Hermansen [24] depicted that maximal oxygen uptake increased almost rectilinearly from the age of 11 to the age of 16 years in males wherefrom it increased at a lower rate reaching a peak value at an age of approximate 25 years. Thereafter, a steady gradual decrease of it was observed. Similar decrease of maximal oxygen uptake with advancement of age was also observed among the lumber jacks [25] who reported a mean VO_2max_ of 48 mL/kg/min at the age group of 22–36 years and decreases up to about 40 mL/kg/min in the age group of 51–66 years. Studies on miners [26] also found a steeper drop with advancement of age where the mean VO_2max_ decreases from 3.4 L/min to 2.5 L/min in the age groups of 21–30 to 31–40 years, respectively. However, the data as reported by Ayoub et al. [13] did not show a steep decline in these occupational personnel. A similar decrease in VO_2max_ in the present study between miners of <40 years and ≥40 years of age group corroborated to the result of earlier studies; in fact altogether the older group showed a decline as compared to the younger age group which was due to the declining capacity in attaining the maximal heart rates and the falling efficiency of circulatory regulation during exercise. Deterioration of the respiratory functional capacity with growing age could have been another attributing factor [22]. 

The direct relation of maximum oxygen uptake with body weight as reported by different authors [8, 27–29] was also well established in this study. The correlation coefficients between these two variables were found to be 0.57 and 0.68 for younger and older age groups at a high level of significance (Table 5). These values were comparable to that reported by Buskrik and Taylor (*r* = 0.63) [30] and Katch (*r* = 0.53) [31], respectively, but lower than that reported by Astrand [27].

## 5. Conclusion

In any occupation, for work stress evaluation, rationalization of workload in terms of acceptable work limit is of prime necessity to safeguard the health of the workers. In the study, carriers showed maximal functional capacities with the least values found in shovelers. Regression equations provide a scope in predicting VO_2max_ in these occupational groups as well. Therefore, the knowledge of a comparatively lower maximal functional capacities of the Indian miners engaged in different activities may be of great importance to determine the fitness criteria of personnel for working in this industry on the perspective of the tolerable limits of work.

## Figures and Tables

**Table 1:** Distribution of subjects in relation to age and different mining activities.

Category	Age group		Total
	<40 yrs	≥40 yrs	
Shoveling	20	17	37
Carrying	21	18	39
Drilling	6	16	22

Total	47	51	98

**Table 2:** Physical characteristics of the subjects.

Parameters	Shoveller *n* = 37	Carrier *n* = 39	Driller *n* = 22	*f* value	*P* value
Age (yr)	41 ± 9.9 (25–58)	39.7 ± 9.6 (23–57)	44.9 ± 8.2 (30–58)	2.19	0.12
Height (cm)	163 ± 7 (147.5–176)	162 ± 5.9 (148.5–178)	163.6 ± 5.6 (151–171.5)	0.51	0.60
Weight (kg)	54.7 ± 7.6 (41–68.5)	53 ± 6.3 (43–68.5)	57.9 ± 8.2 (45–71.5)	3.21	0.04
Body mass index (kg/m^2^)	20.6 ± 2.4 (16–25.9)	20.2 ± 2.1 (16.7–26.2)	21.5 ± 2.5 (17.3–26)	2.09	0.13
Body surface area (m^2^)	1.6 ± 0.1 (1.3–1.8)	1.5 ± 0.1 (1.3–1.8)	1.6 ± 0.1 (1.3–1.8)	2.0	0.14
Experience (yr)	18.4 ± 8.2 (5–33)	19.3 ± 9.5 (5–38)	22.7 ± 8.2 (9–34)	1.76	0.18

Mean ± sd (range).

**Table 3:** Physiological characteristics of the subjects.

Parameters	Shoveller *n* = 37	Carrier *n* = 39	Driller *n* = 22	*f* value	*P* value
HR_rest_ (Beats/min)	66.4 ± 6.9 (52–76)	64.5 ± 6 (52–76)	68.2 ± 5.2 (58–78)	2.69	0.07
HR_max_ (Beats/min)	179 ± 9.9 (162–195)	181 ± 9.3 (163–197)	175 ± 8.2 (162–190)	2.19	0.12
HRR (Beats/min)	113 ± 14.6 (89–137)	117 ± 14.0 (91–143)	107 ± 11.4 (91–125)	2.98	0.06
Absolute VO_2max_ (L/min)	1.89 ± 0.2 (1.54–2.35)	2.03 ± 0.19 (1.64–2.6)	2.08 ± 0.2 (1.65–2.48)	7.19	0.001
Relative VO_2max_ (mL/kg/min)	35 ± 4.6 (28.2–45.7)	38.3 ± 3.7 (30.3–45.6)	36.2 ± 3.3 (31.3–43.1)	6.88	0.002

Mean ± sd (range).

**Table 4:** Relationship between VO_2max_ (mL/kg/min) and age.

Age groups	Equation	*r*	*R^2^*	SE	*t*	*P*
<40 yrs (*n* = 47)	*y* = −0.552*x* + 56.23	−0.55	0.3	3.55	−4.41	<0.001
≥40 yrs (*n* = 51)	*y* = −0.23*x* + 46.28	−0.33	0.11	3.41	−2.44	<0.05

**Table 5:** Relationship between VO_2max_ (L/min) and body weight in two age groups.

Age groups	Equation	*r*	*R^2^*	SE	*t *	*P *
<40 yrs (*n* = 47)	*y* = 0.015*x* + 1.26	0.57	0.33	0.16	4.7	<0.001
≥40 yrs (*n* = 51)	*y* = 0.020*x* + 0.84	0.68	0.46	0.16	6.4	<0.001
